# The Amsterdam Foot Model: a clinically informed multi-segment foot model developed to minimize measurement errors in foot kinematics

**DOI:** 10.1186/s13047-022-00543-6

**Published:** 2022-06-07

**Authors:** Wouter Schallig, Josien C. van den Noort, Marjolein Piening, Geert J. Streekstra, Mario Maas, Marjolein M. van der Krogt, Jaap Harlaar

**Affiliations:** 1 Amsterdam UMC location Vrije Universiteit Amsterdam, Rehabilitation Medicine, De Boelelaan 1117, Amsterdam, The Netherlands; 2 Amsterdam Movement Sciences, Rehabilitation & Development, Amsterdam, the Netherlands; 3 Amsterdam UMC location University of Amsterdam, Radiology and Nuclear Medicine, Meibergdreef 9, Amsterdam, the Netherlands; 4 Amsterdam Movement Sciences, Musculoskeletal Health, Amsterdam, the Netherlands; 5 Amsterdam UMC location University of Amsterdam, Biomedical Engineering and Physics, Meibergdreef 9, Amsterdam, the Netherlands; 6 Department of Biomechanical Engineering, Delft University of Technology, Delft, the Netherlands; 7 Department of Orthopedics & Sports Medicine , ErasmusMC, Rotterdam, the Netherlands

**Keywords:** Foot and ankle kinematics, Gait analysis, Soft tissue artifacts, Marker placement, Repeatability, Validity, Biomechanics

## Abstract

**Background:**

Foot and ankle joint kinematics are measured during clinical gait analyses with marker-based multi-segment foot models. To improve on existing models, measurement errors due to soft tissue artifacts (STAs) and marker misplacements should be reduced. Therefore, the aim of this study is to define a clinically informed, universally applicable multi-segment foot model, which is developed to minimize these measurement errors.

**Methods:**

The Amsterdam foot model (AFM) is a follow-up of existing multi-segment foot models. It was developed by consulting a clinical expert panel and optimizing marker locations and segment definitions to minimize measurement errors. Evaluation of the model was performed in three steps. First, kinematic errors due to STAs were evaluated and compared to two frequently used foot models, i.e. the Oxford and Rizzoli foot models (OFM, RFM). Previously collected computed tomography data was used of 15 asymptomatic feet with markers attached, to determine the joint angles with and without STAs taken into account. Second, the sensitivity to marker misplacements was determined for AFM and compared to OFM and RFM using static standing trials of 19 asymptomatic subjects in which each marker was virtually replaced in multiple directions. Third, a preliminary inter- and intra-tester repeatability analysis was performed by acquiring 3D gait analysis data of 15 healthy subjects, who were equipped by two testers for two sessions. Repeatability of all kinematic parameters was assessed through analysis of the standard deviation (σ) and standard error of measurement (SEM).

**Results:**

The AFM was defined and all calculation methods were provided. Errors in joint angles due to STAs were in general similar or smaller in AFM (≤2.9°) compared to OFM (≤4.0°) and RFM (≤6.7°). AFM was also more robust to marker misplacement than OFM and RFM, as a large sensitivity of kinematic parameters to marker misplacement (i.e. > 1.0°/mm) was found only two times for AFM as opposed to six times for OFM and five times for RFM. The average intra-tester repeatability of AFM angles was σ:2.2[0.9°], SEM:3.3 ± 0.9° and the inter-tester repeatability was σ:3.1[2.1°], SEM:5.2 ± 2.3°.

**Conclusions:**

Measurement errors of AFM are smaller compared to two widely-used multi-segment foot models. This qualifies AFM as a follow-up to existing foot models, which should be evaluated further in a range of clinical application areas.

**Supplementary Information:**

The online version contains supplementary material available at 10.1186/s13047-022-00543-6.

## Background

The dynamic evaluation of clinical foot and ankle disorders is regularly performed with three-dimensional gait analyses including a marker-based multi-segment foot model (MFM). These analyses have been applied in multiple patient populations such as cerebral palsy [1], clubfeet [2], rheumatoid arthritis [3], osteoarthritis [4], diabetes [5], posterior tibial tendon dysfunction [6] and several hallux deformities [7, 8]. In clinical practice these analyses are mainly used to inform and evaluate treatment decisions.

Nearly 40 MFMs have been developed and reviewed [9–13]. The models vary in number of segments, marker sets, anatomical segment definitions and tracking markers. The Oxford foot model (OFM) [14] and Rizzoli foot model (RFM) [15, 16] are among the most frequently used MFMs [9]. These models have been of great value for the scientific community and clinical gait laboratories to provide insight into individual foot kinematics during gait. The repeatability of MFM kinematics has been thoroughly investigated in both healthy and pathological feet [14, 17–22]. However, validation studies of MFMs are limited, as multiple review papers pointed out [9, 10, 13, 23]. This is partly due to the fact that it is challenging to directly measure intrinsic foot bone motion, which acts as a gold standard for marker-based foot models. While intracortical bone pins or emerging dynamic imaging techniques like biplanar videoradiography may enable this [24–26], both are invasive and not widely accessible.

The validity of models can be indirectly assessed by evaluating the sources of measurement errors. Anatomical marker misplacement and soft tissue artifacts (STAs) are considered the two main sources of error that affect the segment coordinate systems (CSs) and consequently the joint kinematics [27, 28]. Especially for multi-segment foot kinematics, measurement errors are more likely to be substantial compared to more proximal body segments, since inter-marker distances are much smaller.

STAs are the movements of tracking markers with respect to their corresponding bony landmarks [28]. In MFMs, largest STAs have been shown for the markers proximally on the posterior aspect of the calcaneus and on the lateral malleolus [29, 30]. The navicular marker has also been shown to have high STAs in some studies [31], however others report relatively small STAs for the navicular marker [30, 32, 33]. STAs cause clinically relevant errors in multi-segment foot kinematics (i.e. > 5°) [24, 30], which are likely to be reduced when tracking markers with large STAs are not included in the kinematic model.

Inconsistent anatomical marker placement by examiners is another source of measurement error and affects mainly the repeatability and interpretation of kinematic data [27]. Studies on the effect of individual marker misplacement on multi-segment foot kinematics (i.e. marker placement sensitivity) identified several anatomical marker locations used in OFM and RFM for which consistent placement is most critical [34–36]. Every segment of OFM and RFM has at least one marker with a placement sensitivity of ≥1°/mm [36]. Since marker placement variability is around 5 mm [37–39], this potentially results in clinically relevant errors. Foot kinematics were mainly sensitive to misplacement of those markers that individually identify the direction of an axis of a local reference frame [36]. This might be improved by taking the midpoint between markers instead of a single one to define an axis of a segment CS.

In addition to the biomechanical validity of the output of a marker model, it should also be clinically meaningful. For a successful clinical application, the model definitions and output need to be interpretable and align with clinical perception. For example, the number of segments, the underlying bones included and their anatomical definitions should correspond to the foot joint motions that are of interest for the clinical problem. Hence, it is important to also include these considerations in the development of a foot model.

To our knowledge, none of the previously-developed MFMs were focused on reducing measurement errors, while these errors are substantial and clinically relevant [30, 36]. Reducing these errors is an important next step in the development of multi-segment foot kinematic measurements. Therefore, the primary aim of this work is to define a multi-segment foot model, called the Amsterdam Foot Model (AFM), that is clinically informed and developed to minimize the errors in kinematic measurements. The secondary aims are to determine the STAs and marker placement sensitivity of AFM compared to OFM and RFM and to explore the inter- and intra-tester repeatability of AFM.

## Methods

### Expert panel

Prior to defining the foot model, a clinical expert panel (see: acknowledgements) was consulted to help align the model to the clinical perception. The panel consisted of 12 experts from several centers in the Netherlands and Belgium. All were professionally active in the field of rehabilitation, physical therapy, orthopedics, radiology, traumatology or human movement sciences, with a special interest in foot and ankle problems. Several one-to-one discussions about their view on foot kinematics were followed up by a joint meeting, to identify which foot kinematic parameters are most informative for their patient populations (e.g. cerebral palsy, clubfoot, Charcot Marie Tooth disease). The discussion was focused on the optimal selection and definition of foot segments and joint angles, in order to maximize the clinically relevant information but avoiding information overload.

### Amsterdam foot model definitions

The definitions of AFM (i.e. marker locations, segment definitions) were based on a combination of knowledge of previously published MFMs, the input of the clinical expert panel and the results of our previous studies on the effect of STAs and marker misplacements on multi-segment foot kinematics [30, 36]. The rationales for the marker placement, the included segments and the individual segment definitions are briefly explained below.

#### Marker placement

The markers (Table 1) are attached while the subject is seated with feet flat on the floor, if possible, and thus in a semi-weightbearing position. This choice was made because it is a compromise between reducing soft-tissue artefacts due to foot loading [30] and allowing accurate palpation of bony landmarks, sometimes requiring the tester to lift and move the foot, which is not possible in standing position for all patients. The marker locations on the foot are the same as in RFM [15, 16].

**Table 1:** Marker set of the Amsterdam Foot Model

Segment	Marker	Abbreviation	Specific location
Shank	1	TT: Tibial tuberosity	Most anterior prominence of the tibial tuberosity
	2^a^	FH: Fibular head	Most proximal apex of the fibular head
	3^b^	ASHN: Anterior shin	Halfway the shank in the center of the tibia
	4^b^	LSHN: Lateral shin	On the line between FH and LM at the height of ASHN
	5^a^	LM: Lateral malleolus	Distal apex of the lateral malleolus
	6^a^	MM: Medial malleolus	Distal apex of the medial malleolus
Hindfoot	7^a^	CALP: Proximally on posterior aspect of calcaneus	Proximally on the midline of the calcaneus posterior aspect (i.e. Achilles’ tendon attachment)
	8	CALD: Distally on posterior aspect of calcaneus	Distally on the midline of the calcaneus posterior aspect
	9	ST: Sustentaculum tali	Most medial apex of the sustentaculum tali
	10	PT: Peroneal Tuberculum	Most lateral apex of the peroneal tubercle
Midfoot	11	NAV: Tuberosity Navicular	Most medial apex of the navicular tuberosity
Forefoot	12	BM1: Base Metatarsal 1	Most proximal point of the 1st metatarsal, at the dorso-medial aspect at approx. 45°
	13	BM5: Base Metatarsal 5	Most proximal point of the 5th metatarsal, at the dorso-medial aspect at approx. 45°
	14	HM1: Head metatarsal 1	Most distal point of the 1st metatarsal, at the dorso-medial aspect at approx. 45°, next to the hallux tendon.
	15	HM5: Head metatarsal 5	Most distal point of the 5th metatarsal, at the dorso-medial aspect at approx. 45°
	16	BM2: Base metatarsal 2	Most proximal point of the 2nd metatarsal
	17	HM2: Head metatarsal 2	Most distal point of the 2nd metatarsal
		*mBM12*	*Midpoint between BM1 and BM2*
		*mBM25*	*Midpoint between BM2 and BM5*
		*mHM12*	*Midpoint between HM1 and HM2*
		*mHM25*	*Midpoint between HM2 and HM5*
Hallux	18	HLX: Proximal phalanx	Most distal point of the proximal phalanx, at the dorso-medial aspect at approx. 45°

Markers with an ^a^ are only used in the static trial as anatomical markers and may therefore be removed in the dynamic trials

Markers with an ^b^ are only used as tracking markers, all unmarked markers are used as anatomical and tracking marker

Markers in italics without a number are virtual markers

#### Segments

**Fig. 1:**
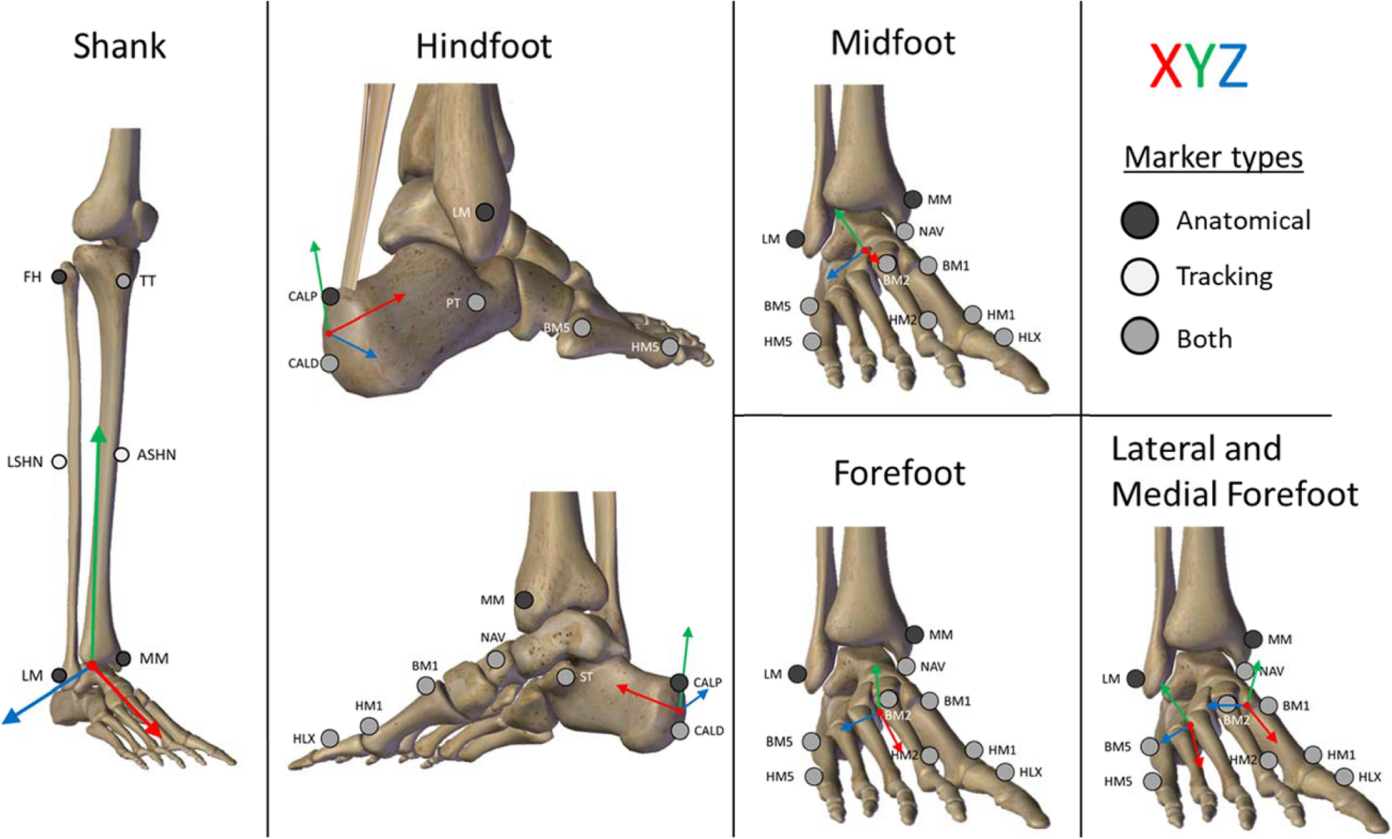
Schematic overview of the marker placement and anatomical coordinate systems of the different segments of the Amsterdam Foot Model. The bone anatomy images are created using ZygoteBody Professional™ zygotebody.com

The AFM consists of 5, and optionally 6 segments: shank, hindfoot, midfoot, forefoot (optionally divided into a medial and lateral forefoot) and hallux. All segments are assumed to be rigid. The definition of the anatomical CS of each segment is described in Fig. 1 and Table 2. Anatomical CSs are defined to represent the bony structures in the foot and are used to calculate the joint angles (i.e. rotation between the anatomical CS of two segments). Following ISB recommendations [40], for all segments of both feet the x-axis is pointing forward, the y-axis is pointing upward and the z-axis is pointing to the right when the subject is in anatomical position. Additionally, technical tracking CSs are defined for the shank, hindfoot and forefoot. These CSs are used to track the foot segment during gait. Anatomical CSs are expressed in the technical CSs during a static trial, to be able to reconstruct the anatomical CS during gait and subsequently calculate joint angles.

**Table 2:** Definitions of the segment anatomical coordinate systems of the Amsterdam Foot Model for the right foot

*Segment*		*Description*	*Formula*
**Shank**^a^	*Origin*	Midpoint between LM and MM	O = (LM + MM)/2
	*Anterior-Posterior axis (x)*	⊥ to the plane defined by Y and the vector from LM to origin	X = cross(Y, LM-O^c^)
	*Vertical axis (y)*	From origin to projection of TT on the plane defined by the origin, LM and FH	TT_proj = projection (TT,X,LM)Y = TT_proj-O
	*Medio-Lateral axis (z)*	⊥ to the plane defined by X and Y	Z = cross(X,Y)
**Hindfoot**	*Origin*	Midpoint between CALD and CALP	O = (CALD+CALP)/2
	*Anterior-Posterior axis (x)*	From Origin to midpoint ST and PT	X = (ST + PT)/2-O
	*Vertical axis (y)*	⊥ to the plane defined by Z and X	Y = cross(Z,X)
	*Medio-Lateral axis (z)*	⊥ to the plane defined by X and the vector from CALD to CALP.	Z = cross(X,CALP-CALD)
**Midfoot**^b^	*Origin*	Midpoint between NAV and BM5	O = (NAV + BM5)/2
	*Anterior-Posterior axis (x)*	From origin to BM2	X = BM2-O
	*Vertical axis (y)*	⊥ to the plane defined by NAV, BM2, BM5	Y = cross (BM5-NAV^c^,BM2-NAV)
	*Medio-Lateral axis (z)*	⊥ to the plane defined by X and Y	Z = cross(X,Y)
**Forefoot**	*Origin*	Midpoint between mBM12 and mBM25	O = (mBM12 + mBM25)/2
	*Anterior-Posterior axis (x)*	From origin to midpoint mHM12 and mHM25	X = (mHM12 + mHM25)/2-O
	*Vertical axis (y)*	⊥ to the plane defined by origin, mHM12 and mHM25	Y = cross (mHM25-O^c^,mHM12-O)
	*Medio-Lateral axis (z)*	⊥ to the plane defined by X and Y	Z = cross(X,Y)
**Medial Forefoot**	*Origin*	mBM12	O = mBM12
	*Anterior-Posterior axis (x)*	From origin to mHM12	X = mHM12-O
	*Vertical axis (y)*	⊥ to the plane defined by X and the vector from BM1 to BM2	Y = cross (BM2-BM1^c^,X)
	*Medio-Lateral axis (z)*	⊥ to the plane defined by X and Y	Z = cross(X,Y)
**Lateral Forefoot**	*Origin*	mBM25	O = mBM25
	*Anterior-Posterior axis (x)*	From origin to mHM25	X = mHM25-O
	*Vertical axis (y)*	⊥ to the plane defined by X and the vector from BM2 to BM5	Y = cross (BM5-BM2^c^),X)
	*Medio-Lateral axis (z)*	⊥ to the plane defined by X and Y	Z = cross(X,Y)
**Hallux**	*Origin*	HM1	O=HM1
	*Anterior-Posterior axis (x)*	From origin to HLX	X = HLX-O
	*Vertical axis (y)*	⊥ to the plane defined by X and Z of the medial forefoot	Y = cross (Zmedial_forefoot,X)
	*Medio-Lateral axis (z)*	⊥ to the plane defined by X and Z	Z = cross(X,Z)

*NOTE:* cross (A,B) calculates the cross product between A and B; projection (Marker,Norm Vector,Point) calculates the projection of Marker on a plane with its Norm Vector and a Point on that plane. ⊥ = perpendicular. ^a^ As in Cappozzo et al. (1995) ^b^ As in Leardini et al. (2007). ^c^When the calculation are performed for the left foot the order of the subtraction is changed. Markers abbreviations are explained in Table 1

##### Shank

The shank segment consists of the tibia and fibula. Its anatomical definition is based on Cappozzo et al. [41]. Its technical CS is created with TT, ASHN and LSHN (see Table 1 for full marker names), which does not include the lateral malleolus marker because of its large STAs [30], nor does it include the medial malleolar marker because it gets knocked off easily during gait, especially in some pathological gait patterns. TT was chosen over the fibular head because it has smaller STAs [42].

##### Hindfoot

The hindfoot segment consists of the calcaneal bone. For its anatomical CS, the midpoint between the CALD and CALP marker is used, so the CS is more robust to marker misplacement. In addition, using the midpoint results in a slightly upward rotated anterior axis in neutral foot position. This aligns more closely with the calcaneal pitch, which is a frequently used radiographic measure used in clinical practice when evaluating the hindfoot. Furthermore, CALD and CALP were used to define the vertical axis of the hindfoot, because the varus-valgus angle of the hindfoot is considered as a clinically relevant angle, which is best described by the two markers at the posterior aspect of the calcaneus. To track the hindfoot coordinate system, the CALP marker was not used because it demonstrated very large STAs [30], hence CALD, ST and PT are used as tracking markers.

##### Midfoot

The midfoot segment consist of the navicular, cuboid and cuneiform bones. Defining a midfoot segment allows for measuring Chopart and Lisfranc joint motion. Measuring these joints separately was preferred by the clinical expert panel, because some patient populations have complaints at these joints. The midfoot has limited marker placement possibilities, hence it was chosen to use the same anatomical and technical CS as in RFM, which are based on the NAV, BM2 and BM5 markers [15].

##### Forefoot

The forefoot segment consist of the 5 metatarsal bones. The anatomical CS is defined by the midpoints between BM1 and BM2, HM1 and HM2, BM2 and BM5, HM2 and HM5 to make the CS more robust to marker misplacements. To determine the tracking marker for the forefoot, the STAs were minimized with the constraint of one marker on the 1st, 2nd and 5th metatarsal, so the whole forefoot was tracked, resulting in BM1, HM2 and BM5 as tracking markers.

##### Medial and lateral forefoot

The rigid body assumption is the least valid for the forefoot segment [43]. Moreover, our expert panel expressed a strong wish to be able to be informed about the deformation of the forefoot, by distinguishing between a medial and lateral part with an associated axis roughly through the second metatarsal. Hence, AFM provides the option to separately measure the movements of the medial and lateral forefoot. The medial forefoot consists of the 1st and 2nd metatarsal and the lateral forefoot consist of the 2nd until the 5th metatarsal. For the anatomical CSs, the same midpoints between the respective metatarsal markers were used as in the forefoot segment to improve the sensitivity to marker misplacements.

##### Hallux

The hallux segment describes the proximal phalanx, using a single marker that is placed on the distal aspect of the phalanx. We chose not to use a marker triad because it is easily kicked off, especially in gait patterns without a proper foot clearance. The hallux-related angles are not calculated as planar angles because of mathematical singularities that arise in the trigonometric calculation when large rotations take place [16]. Therefore, a hallux CS is created based on the vector from HM1 to HLX and the medio-lateral axis of the medial forefoot segment around which it rotates. Forefoot markers are also used to define the anatomical coordinate system of the hallux segment in the modified version of RFM [16]. However, as only a single marker is placed on the hallux, only motions in the sagittal and transverse plane can be calculated.

#### Joint angles

Kinematics were calculated for the following seven joints: hindfoot-shank (HF-SK, i.e. talocrural and subtalar joints), midfoot-hindfoot (MF-HF, i.e. Chopart joint), forefoot-hindfoot (FF-HF), forefoot-midfoot (FF-MF, i.e. Lisfranc joint), lateral forefoot-midfoot (FF_L_-MF, i.e. lateral Lisfranc joint), medial forefoot-midfoot (FF_M_-MF, i.e. medial Lisfranc joint), hallux-medial forefoot (HX-FF_M_, i.e. MTP joint) and foot-shank (F-SK) for which the foot segment definitions of the applied lower extremity marker model can be used. Depending on the clinical or research question a subset of these joint kinematics can be calculated.

All joint kinematics are expressed as a decomposition into three sequential rotations between two segments according to ISB definitions [40] and Grood and Suntay [44]. This means that the first rotation is around the medio-lateral (z) axis of the proximal segment, resulting in dorsal and plantar flexion. The second rotation is around the floating anterior-posterior (x) axis, which is termed inversion and eversion. The third rotation is around the superior-inferior (y) axis of the distal segment, defined as internal- and external rotation at the ankle joint and adduction and abduction at the more distal joints.

#### Planar angles

In addition, the medial longitudinal arch (MLA) and the transverse tarsal arch (TTA) are calculated as planar angles. These arches are a representation of the midtarsal joint poses due to active and passive foot structures, and are believed to play a major role in stiffening the foot during gait [45]. The medial longitudinal arch is defined as the angle between a proximal and distal 3D vector. First, in the static trial, a virtual calcaneus marker is created by taking the midpoint between CALP and the midpoint between ST and PT, which is then projected on the ground (CAp). The first vector is from CAp to the navicular marker. The second vector is from the head of the 1st metatarsal projected (HM1p) on the ground to the navicular marker. The projected marker positions (CAp and HM1p) are fixed to the corresponding segment CS (i.e. hindfoot or forefoot) for calculations in the dynamic trials. This MLA definition has been shown to be most accurate compared to other MLA definitions [46]. However, it can only be used when complete foot contact is present in the static trial, otherwise the projected markers are not at the sole of the foot as intended. In some patient populations (e.g. cerebral palsy with a rigid equinus deformity) it is not always possible to stand with complete foot contact. In that case. The average position of the virtual CAp marker in the hindfoot coordinate system of a reference dataset is used, which is normalized to calcaneal length (i.e. distance between the CALP marker and the midpoint between the ST and PT marker). TTA is defined as the angle between the vector between BM1 and BM2 and the vector between BM2 and BM5.

### Model evaluation

The evaluation of AFM was performed in three parts. Two sources of measurement errors were evaluated: 1) soft tissue artifacts and 2) marker placement sensitivity. In addition, 3) the intra- and inter-tester repeatability of the foot kinematics as calculated by AFM were explored. All data analyses were performed using Matlab (R2017b, MathWorks, USA). The measurement errors due to soft tissue artifacts and marker placement sensitivity of OFM and RFM have been published previously [30, 36] and are included in this study as comparison to AFM.

#### Soft tissue artifacts

The used methodology to quantify STAs has been described in detail previously [30]. In brief, fifteen subjects with an asymptomatic right foot and ankle were included (8 females, 24.9 ± 1.8 years, 176.7 ± 7.5 cm, 73.2 ± 12.1 kg, EU shoe size: 40.9 ± 2.2). A marker set that included all AFM, OFM and RFM markers was placed on the right foot. Subjects were seated on a custom-made loading device [47]. A total of 10 computed tomography (CT) scans were performed, including one unloaded reference scan with neutral foot position and 9 loaded scans with the foot in a variety of positions, ranging from 40° plantar flexion (40PF) to 20° dorsal flexion (20DF) and from 10° inversion (10INV) to 10° eversion (10EV) to gain insight in STAs in different foot positions.

Custom-made software was used to process the CT-scans [48]. In the first unloaded scan, a segmentation was performed of all 24 markers and 10 underlying bones. Subsequently, these segmented objects were registered (i.e. matched) to the other scans to obtain their position and orientation in all scans [49]. The 3D displacement of each marker with respect to its underlying bone (i.e. STA) was quantified for all foot positions [30]. Next, the segment orientation and joint angles according to AFM, OFM and RFM definitions were determined based on the marker positions with and without STAs, to assess their kinematic errors due to STAs [30]. The data for OFM and RFM has already been presented elsewhere [30] and is used in the current study as a comparison.

Errors in segment orientations (shank, hindfoot, forefoot) and joint angles (hindfoot-shank, forefoot-hindfoot) were compared between models (AFM, OFM, RFM) and foot plate positions with 2-way repeated measures ANOVAs. When significant (*p* < 0.05), post-hoc analyses with Bonferroni correction (for the 7 foot positions per angle in each plane) were performed in which AFM was compared to OFM and RFM separately.

#### Marker placement sensitivity

The methodology to determine the marker placement sensitivity has been described in detail previously [36]. In short, ten adults (6 females, 26.8 ± 2.6 years, 176.4 ± 8.1 cm, 67.2 ± 8.5 kg, EU shoe size: 41 ± 2, range 38–45) and nine children (5 female, 10.7 ± 1.9 years, 147.7 ± 12.8 cm, 41.1 ± 10.9 kg, EU shoe size: 36 ± 2, range: 31–38) with asymptomatic feet were included. A set of 21 markers was placed on the right foot and shank, which included all AFM, OFM and RFM markers. In addition to the markers presented in Table 1, markers were placed on the medial and lateral side of the calcaneus (MCAL, LCAL) and on the medial side of the head of the first metatarsal (HM1M). Marker positions were recorded by a 12-camera motion capture system (Vicon Motion Systems Ltd., Oxford, UK) during a static upright standing trial. In post processing, each marker was virtually displaced ±1 cm, in steps of 1 mm, over the anterior-posterior (x), superior-inferior (y) and medio-lateral (z) axis of a foot-specific CS as defined according to Cappozzo et al. [41]. For every displacement the segment CS was determined as well as its orientation with respect to the reference pose in which no markers were displaced. For each displacement direction, a linear fit was made over the angular error in one of three planes between − 10 and 10 mm displacement. The gradient of this line described the sensitivity of the segment orientation to marker misplacement in °/mm.

#### Repeatability

Fifteen healthy participants (9 females, age: 26.7 ± 2.9 years, height: 173.7 ± 7.8 cm, weight: 67.1 ± 9.1 kg, EU shoe size: 41 ± 2) were included. Subjects had asymptomatic feet as defined by 1) not wearing insoles, 2) no history of foot and ankle surgeries and 3) no recent (within 3 months) foot and ankle complaints.

Subjects underwent a 3D gait analysis with ø12.7 mm markers on the trunk and lower extremities according to Cappozzo et al. [41] and ø9.5 mm markers according to AFM (Table 1) on their right foot and lower leg. The foot model markers were placed three times during one visit in a semi-weight bearing position. The first and third time by tester A and the second time by tester B. Tester A was blinded for the marker placement of tester B and vice versa. Both testers had three and a half years of clinical experience in placing foot model markers. After each time the markers were placed, a static standing trial and five walking trials were collected. The static trial was performed to calculate the static joint angles and to define the anatomical CSs with respect to the technical CSs for each segment. Walking trials were performed barefoot at comfortable walking speed on a 10 m walkway, until five successful trials were collected. During all trials, marker trajectories were recorded by a 12-camera motion capture system (Vicon Motion Systems Ltd., Oxford, UK) using the Vicon Nexus software (version 2.6.1). The strides were normalized to 100% of the gait cycle, by determining initial contact and toe-off based on the foot velocity [50] and averaged. The range of motion (ROM) was quantified for each trial separately as the difference between the maximum and minimum angle, and subsequently averaged over trials.

Inter- and intra-tester repeatability were determined for all angles. For each percent of the gait cycle the variability was calculated as the standard deviation (SD) between the two measurements of tester 1 (intra-tester) and between tester 2 and the first measurement of tester 1 (inter-tester). These were averaged over the gait cycle to obtain one variability value (σ) for each angle for each participant. The σ for each angle was assessed for normality with the Shapiro-Wilk test and presented as median [interquartile range] when the σ of a considerable part of the angles was not normally distributed. In addition, the standard error of measurement (SEM) was calculated, which equals the square root of the error variance and is a measure of agreement [51]. To obtain insight into the static and dynamic component of the repeatability of the angular gait curves, the same measures (i.e. σ and SEM) were also calculated for the ROM and the joint angles in the static standing trial. To interpret the SEM we follow McGinley et al. [52], who considers errors smaller than 5° as clinically acceptable in lower extremity kinematics.

Data of this repeatability study in adults, as well as a similar dataset for 15 typically developing children (7 females, age: 10.3 ± 3.2 years, height: 144.8 ± 19.4 cm, weight: 37.1 ± 14.0 kg, EU shoe size: 36 ± 5), was made available to serve as a reference dataset for future studies using AFM [53].

## Results

### Soft tissue artifacts

**Fig. 2:**
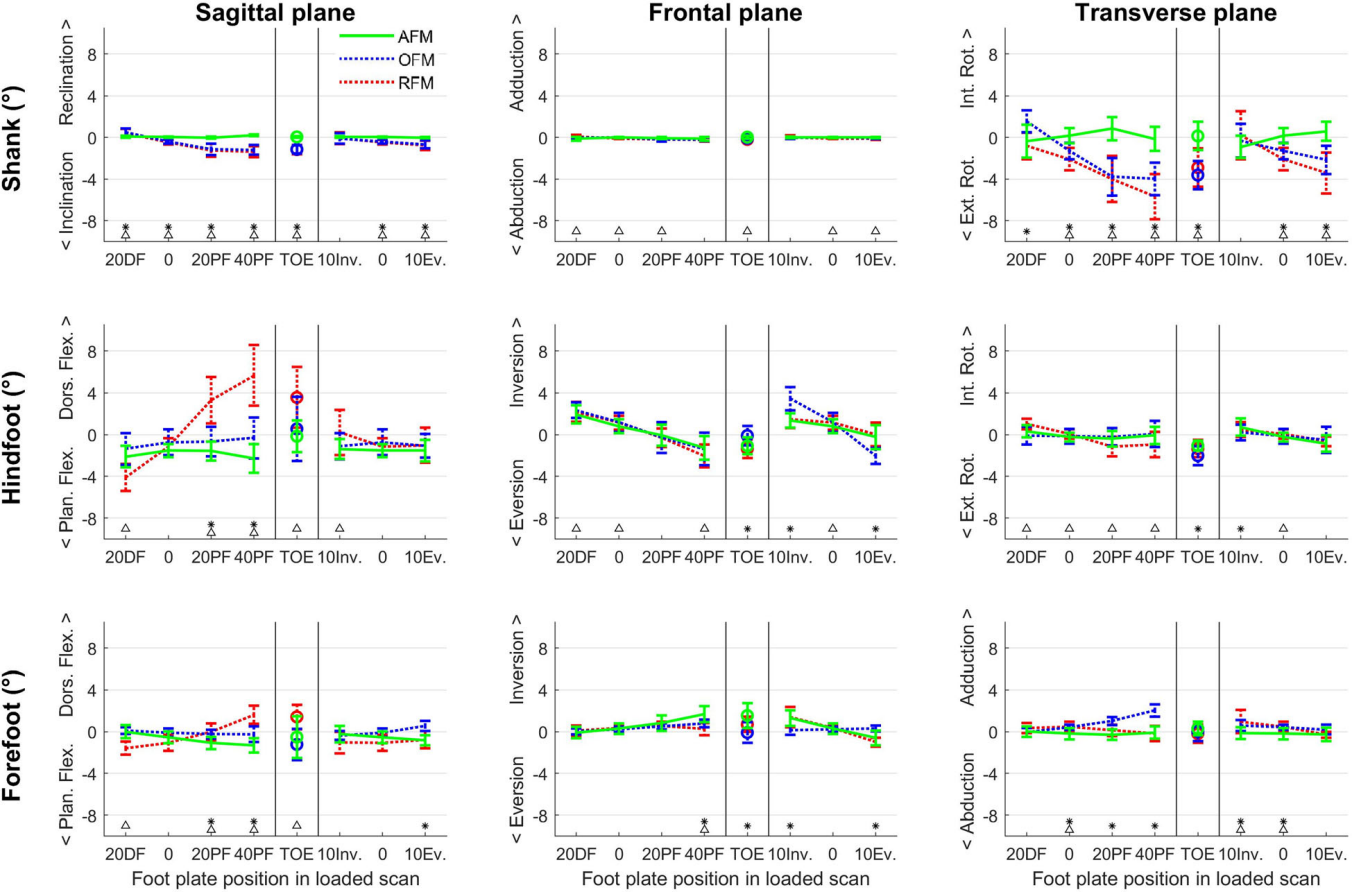
The angular error in segment orientation due to soft tissue artifacts for the shank, hindfoot and forefoot segment of the Amsterdam Foot Model (AFM, black), Oxford Foot Model (OFM, dotted blue) and Rizzoli Foot Model (RFM, dotted red) in the different loaded foot plate positions in three planes. OFM and RFM data is adopted from Schallig et al. [30] as a comparison. * indicates that AFM and OFM are significantly different. ∆ indicates that AFM and RFM are significantly different

**Fig. 3:**
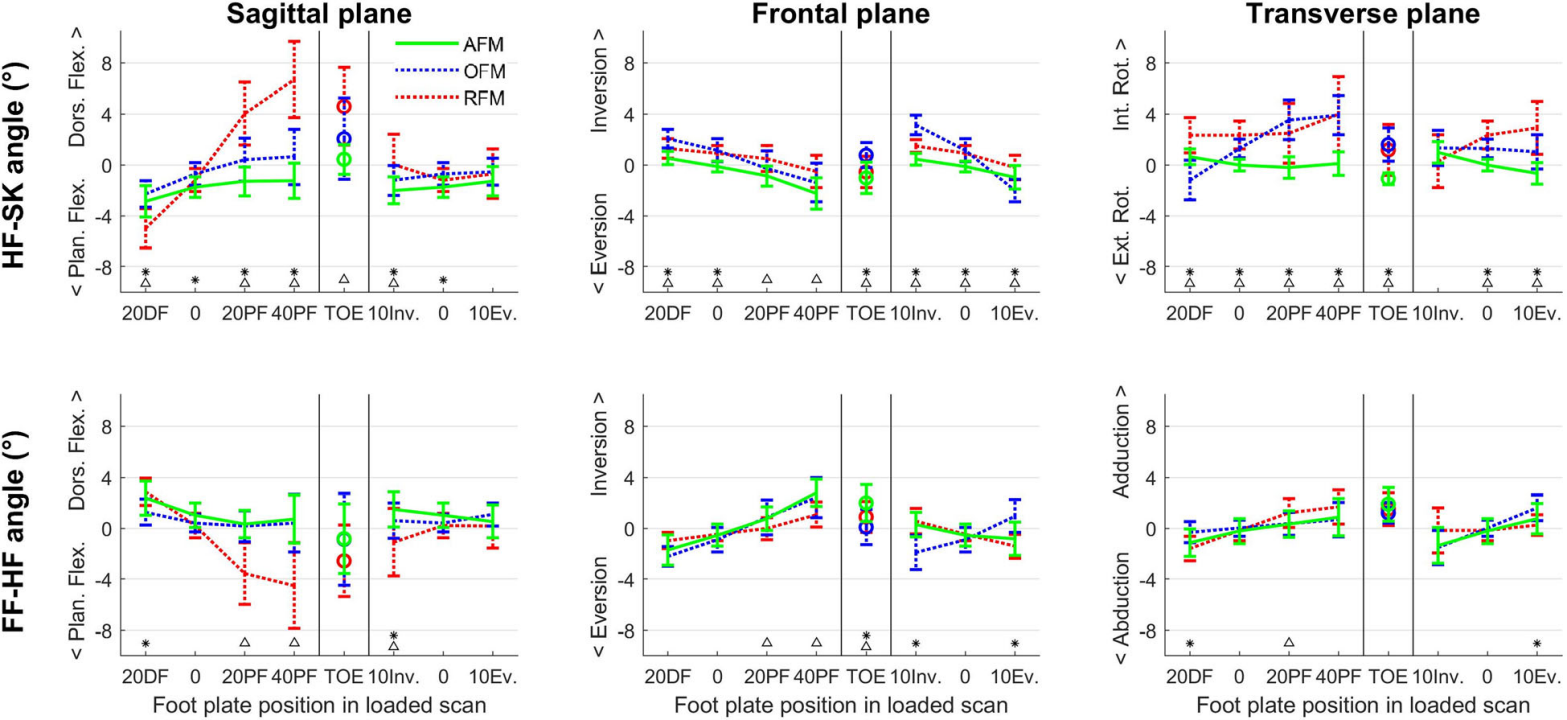
The angular error in joint angles due to soft tissue artifacts for the hindfoot-shank and forefoot-hindfoot angle of the Amsterdam Foot Model (AFM, black), Oxford Foot Model (OFM, dotted blue) and Rizzoli Foot Model (RFM, dotted red) in the different loaded foot plate positions in three planes. OFM and RFM data is adopted from Schallig et al. [30] as a comparison. * indicates that AFM and OFM are significantly different. ∆ indicates that AFM and RFM are significantly different

The effect of STAs on the segment orientations and joint angles as calculated by AFM are presented (Figs. 2 and 3) and compared to previously published data of OFM and RFM [30]. For segment orientations, AFM errors due to STAs were ≤ 0.9° for the shank, ≤2.1° for the hindfoot and ≤ 1.7° for the forefoot. AFM errors for the shank were significantly smaller (i.e. close to zero) compared to OFM (≤4.0°) and RFM (≤5.7°) mainly in the transverse and sagittal plane. For the hindfoot, AFM errors were smaller or similar compared to RFM, with the largest difference in the sagittal plane (AFM:2.1°, RFM:5.7°). AFM and OFM errors were similar except for small differences in the sagittal plane in favor of OFM and in the frontal plane in favor of AFM. For the forefoot, AFM showed slightly smaller errors compared to RFM in the sagittal plane and compared to OFM in the transverse plane, but all were ≤ 2.0°.

For the joint angles, errors in the hindfoot-shank and forefoot-hindfoot angles for AFM were ≤ 2.9°. For the hindfoot-shank angle, the errors were significantly smaller than or similar to the errors of RFM (≤6.7°) except for the frontal plane errors in the plantar flexion foot position. Compared to the errors in OFM (≤3.9°), AFM hindfoot-shank angles were smaller in the frontal and transverse plane, but slightly larger (max. Δ:1.0°) in the sagittal plane for almost all foot positions. For the forefoot-hindfoot angle, mainly in the sagittal plane AFM errors (≤2.4°) were smaller compared to RFM errors (≤4.5°). Only a few small differences (max. Δ:1.3°) were found between AFM and OFM.

### Marker placement sensitivity

**Fig. 4:**
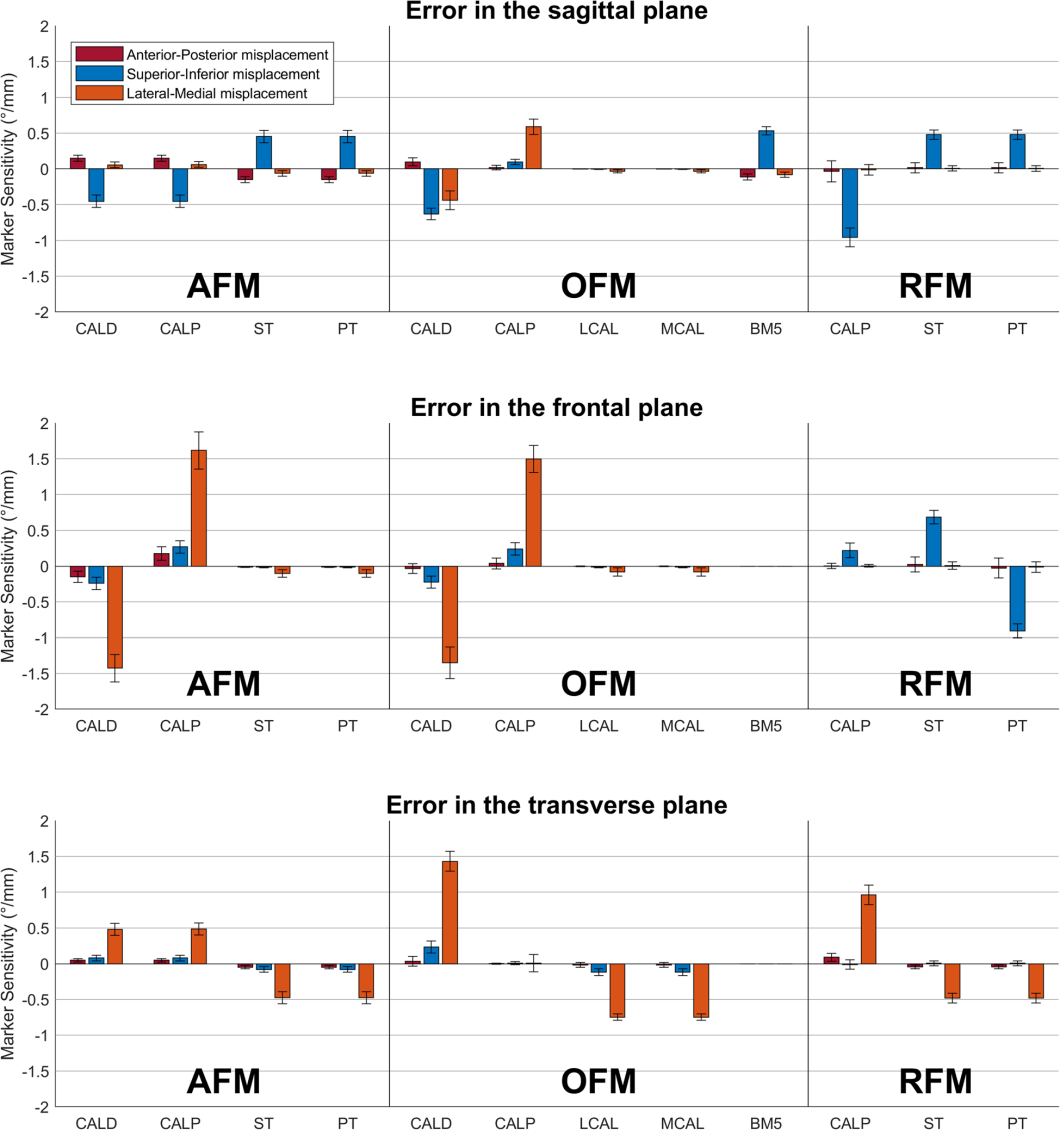
Segment orientation error due to virtual marker misplacements (i.e. marker placement sensitivity) for the hindfoot segment of AFM, OFM and RFM Positive values indicate dorsal flexion, inversion, internal rotation and adduction. Most marker abbreviations are provided in Table 1 and the others in the main text.

**Fig. 5:**
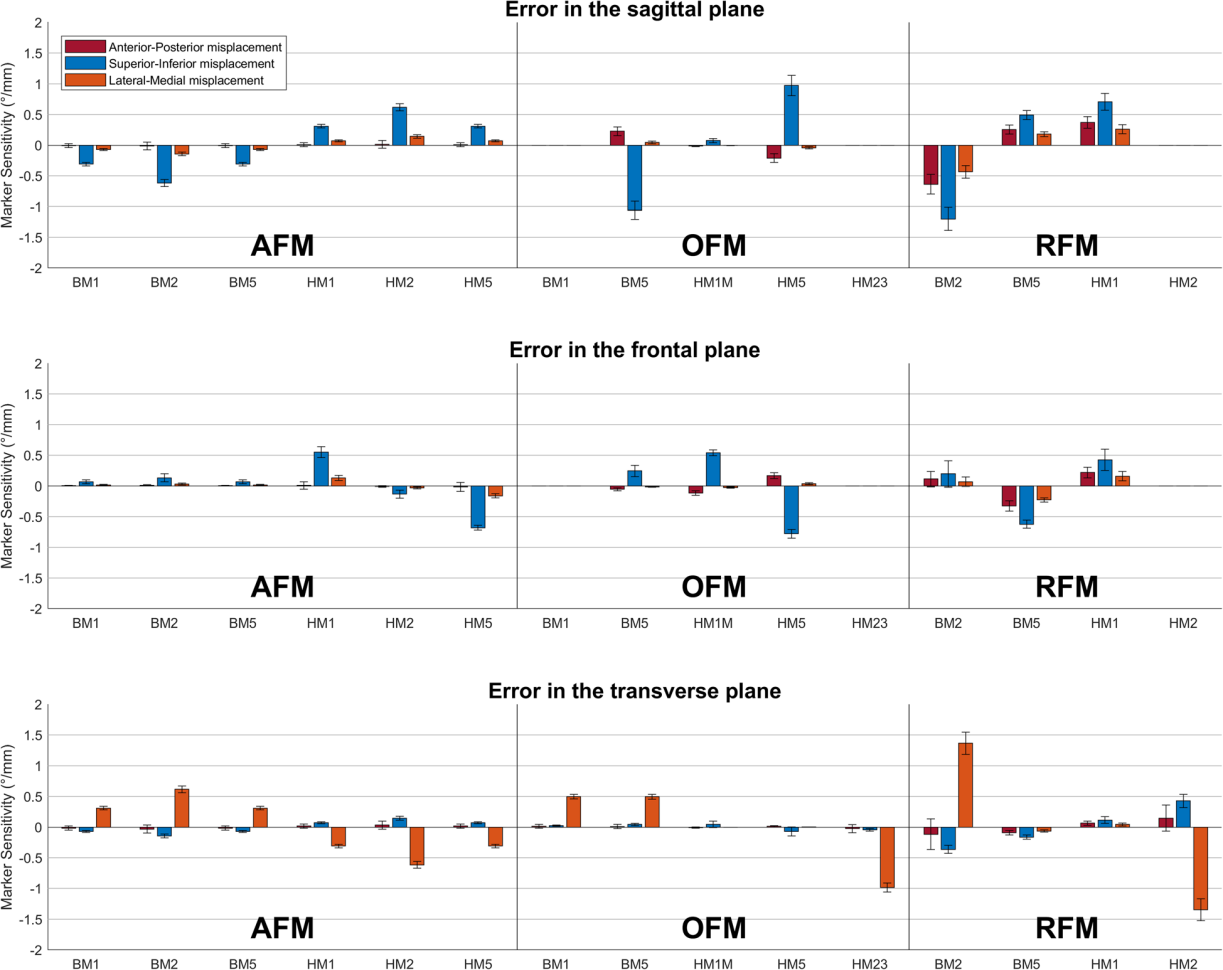
Segment orientation error due to virtual marker misplacements (i.e. marker placement sensitivity) for the forefoot segment of AFM, OFM and RFM. Positive values indicate dorsal flexion, inversion, internal rotation and adduction. Most marker abbreviations are provided in Table 1 and the others in the main text

Marker placement sensitivity of AFM, OFM and RFM is shown for the hindfoot (Fig. 4) and forefoot segment orientation (Fig. 5). OFM and RFM data was adopted from Schallig et al. [36]. The majority of these sensitivity values was < 0.2 °/mm (71% for AFM, 76% for OFM and 59% for RFM). In AFM only 2 large sensitivity values (i.e. ≥1.0 °/mm) were present, compared to six in OFM and five in RFM.

For the hindfoot, all the marker placement sensitivity values of AFM (*n* = 36) were < 0.5°/mm, except two that affected the frontal plane (1.4–1.6 °/mm) when displacing the CALD or CALP marker in medio-lateral direction. Compared to AFM, the other two models showed more values > 0.5 °/mm. OFM presented eight sensitivity values > 0.5 °/mm, with the highest errors (1.4–1.5 °/mm) in the frontal and transverse plane when displacing the markers on the posterior aspect of the calcaneus (CALD, CALP) in medio-lateral direction. For RFM, four sensitivity values > 0.5 °/mm were present, of which the largest were 1.0 °/mm when displacing the CALP marker in superior-inferior or medio-lateral direction.

For the forefoot, AFM showed 6 marker placement sensitivity values > 0.5 °/mm and the other two models 7. Moreover, AFM showed no values of ≥1.0 °/mm, while OFM and RFM both showed 3 values ≥1.0 °/mm. OFM showed errors in the sagittal plane when displacing the markers on the 5th metatarsal in superior-inferior direction and in the transverse plane when displacing the HM2 marker in medio-lateral direction. For RFM, highest sensitivity was shown for displacing the BM2 or HM2 markers (1.2–1.4 °/mm) in medio-lateral or superior-inferior direction.

### Repeatability

The repeatability and agreement (σ and SEM) of AFM for the kinematic temporal patterns, static angles and ROM are shown in Table 3. For 45% of the angles, the variability values were not normally distributed and therefore they are presented as median [IQR]. The median inter-tester variability (σ) and the average SEM over all angles was 3.1[2.1°] and 5.2 ± 2.3° for the curves. The variability in curves was mainly a result of a static offset (σ:2.9[2.4°], SEM:4.8 ± 2.2°), rather than a dynamic component as shown by the lower variability in ROM (σ:0.7[0.5°], SEM:1.4 ± 1.0°). The intra-tester repeatability was in general lower than the inter-tester repeatability and again similar for the curves (σ:2.2[0.9°], SEM:3.3 ± 0.9°) and static angles (σ:2.0[0.8°], SEM:3.2 ± 0.9°) and lower for the ROM (σ:0.6[0.3°], SEM:0.9 ± 0.5°).

**Table 3:** Inter- and intra-tester repeatability of AFM

		Inter-tester						Intra-tester					
		σ (°)			SEM (°)			σ (°)			SEM (°)		
Angle	Plane	Curve	Static	ROM	Curve	Static	ROM	Curve	Static	ROM	Curve	Static	ROM
HF-SK	Sag.	3.5 [1.9]	3.1 [2.4]	0.9 [1.4]	3.6	3.7	1.3	2.1 [1.9]	1.2 [2.7]	1.2 [0.7]	2.5	2.4	1.2
	Front.	2.0 [2.4]	2.4 [3.0]	0.6 [0.8]	4.3	3.7	1.1	2.3 [2.7]	1.9 [3.4]	0.3 [0.4]	5.2	5.2	0.4
	Trans.	3.4 [3.2]	3.5 [2.4]	0.5 [1.5]	4.7	3.8	1.1	1.6 [1.9]	1.7 [1.7]	0.6 [0.7]	2.7	2.5	0.9
FF-HF	Sag.	5.3 [3.5]	3.2 [4.3]	0.6 [0.8]	5.4	4.9	0.8	1.4 [3.3]	2.3 [2.3]	0.6 [0.6]	2.9	2.6	1.0
	Front.	2.7 [6.0]	2.9 [5.8]	1.0 [1.1]	5.5	5.0	1.4	3.3 [2.2]	3.4 [2.1]	0.6 [0.6]	4.8	4.8	0.8
	Trans.	1.9 [3.0]	1.8 [2.0]	1.0 [1.7]	3.2	2.9	1.8	1.2 [2.9]	1.9 [3.1]	0.6 [1.1]	2.8	2.9	0.9
FF-SK	Sag.	2.9 [2.4]	1.5 [2.0]	1.4 [2.2]	3.3	2.6	2.2	2.3 [1.7]	1.1 [1.8]	1.3 [1.0]	2.9	2.6	1.6
	Front.	1.3 [1.1]	0.9 [1.3]	1.2 [1.0]	2.4	1.8	1.5	1.4 [1.2]	1.4 [2.4]	0.7 [0.8]	2.8	2.7	1.0
	Trans.	3.4 [4.1]	4.8 [4.1]	0.9 [1.6]	4.7	4.8	1.8	2.2 [1.3]	1.7 [2.2]	0.4 [0.8]	2.6	2.4	0.8
MF-HF	Sag.	3.6 [4.6]	3.0 [4.5]	1.0 [0.7]	5.6	5.1	1.4	2.5 [2.1]	2.2 [2.1]	0.7 [1.1]	2.9	3.4	1.2
	Front.	2.7 [5.5]	2.4 [4.1]	0.7 [0.7]	5.6	5.2	1.0	2.5 [2.7]	2.0 [1.9]	0.5 [0.6]	4.5	4.4	0.8
	Trans.	5.5 [4.2]	4.8 [3.6]	1.5 [0.8]	6.3	5.5	1.7	1.9 [3.6]	2.3 [3.8]	0.7 [0.6]	3.8	3.8	1.2
FF-MF	Sag.	4.8 [5.3]	5.4 [4.5]	0.5 [0.9]	6.6	6.1	0.8	2.3 [3.0]	1.6 [2.3]	0.4 [0.3]	3.0	2.6	0.7
	Front.	3.3 [3.2]	2.5 [4.4]	0.9 [0.8]	3.4	3.7	1.1	2.4 [2.4]	2.6 [2.7]	0.6 [0.7]	3.1	3.0	0.8
	Trans.	2.7 [3.2]	2.5 [1.9]	0.5 [0.4]	3.5	3.0	0.7	2.0 [1.2]	2.0 [1.6]	0.3 [0.3]	2.9	2.8	0.5
FF_M_-MF	Sag.	6.4 [6.1]	5.7 [5.8]	1.2 [1.1]	7.5	7.0	1.6	3.1 [1.7]	2.3 [1.2]	0.9 [1.0]	4.1	3.3	1.3
	Front.	12.2 [8.7]	11.6 [11.2]	0.5 [0.8]	12.4	11.5	1.0	3.1 [3.6]	3.1 [3.6]	0.4 [0.4]	4.1	4.3	0.4
	Trans.	3.0 [3.6]	3.5 [2.7]	0.4 [0.5]	4.6	3.7	0.7	3.1 [3.4]	2.8 [2.7]	0.2 [0.5]	4.2	3.8	0.6
FF_L_-MF	Sag.	3.1 [2.2]	2.7 [3.1]	0.7 [0.6]	4.4	4.2	1.1	1.9 [1.8]	1.9 [1.4]	0.6 [0.8]	2.2	2.2	0.9
	Front.	2.1 [1.7]	1.9 [2.1]	0.5 [0.8]	3.2	3.1	0.7	1.0 [2.0]	0.9 [1.9]	0.5 [0.5]	1.9	1.9	0.6
	Trans.	2.9 [4.0]	2.1 [3.1]	0.3 [0.5]	4.4	3.6	0.5	2.1 [2.3]	2.2 [2.3]	0.4 [0.3]	3.7	3.3	0.5
HX-FF_M_	Sag.	9.0 [7.0]	6.5 [7.7]	4.2 [1.6]	9.2	8.0	4.6	1.6 [2.3]	1.3 [2.7]	1.1 [1.0]	2.6	2.6	1.4
	Front.	–	–	–	–	–	–	–	–	–	–	–	–
	Trans.	2.8 [1.5]	2.4 [3.1]	3.2 [3.1]	4.0	3.2	4.2	2.9 [2.5]	2.9 [2.6]	2.1 [1.8]	4.0	3.6	2.6
MLA		1.4 [1.9]	1.9 [2.6]	0.4 [0.6]	3.1	6.7	1.0	1.2 [1.5]	1.3 [1.4]	0.4 [0.5]	2.2	2.1	0.8
TTA		10.2 [5.7]	7.1 [7.0]	0.4 [1.1]	9.3	8.6	1.1	2.7 [2.3]	2.8 [2.9]	0.5 [0.8]	4.1	4.4	0.8

For the inter-tester SEM, 75% of all outcomes was clinically acceptable (i.e. < 5°). Especially the joint angles that involved the midfoot or medial forefoot were larger than 5°. For the intra-tester SEM, almost all outcomes (97%) were < 5°, except for the HF-SK angle in the frontal plane (5.2°).

## Discussion

The aim of this study was to define a clinically informed multi-segment foot model that is developed to minimize measurement errors. The resulting Amsterdam Foot Model demonstrated measurement errors due to soft tissue artifacts and marker misplacements that were considerably smaller than the frequently used Oxford and Rizzoli Foot Models and is therefore an improved follow-up to these models.

The dynamic multi-segment foot kinematic measurements are improved compared to conventional MFMs by using tracking markers with relatively small STAs, as recommended by the ISB [23]. Largest STAs have previously been shown for the markers on the lateral malleolus and proximally on the posterior aspect of the calcaneus, mainly causing errors in the transverse plane of the shank segment and the sagittal plane of the hindfoot segment [30]. Both markers were not used as tracking markers in AFM. Consequently, the errors in the shank segment were smaller in AFM (≤0.9°) compared to OFM (≤4.0°) and RFM (≤5.7°) and errors in the joint angles around the hindfoot of AFM in the sagittal plane (≤2.9°) were reduced compared to RFM (≤6.7°), but not compared to OFM (≤2.3°), which already had a small mean error in this plane. The error in the hindfoot-shank angle of AFM in the neutral loaded condition was similar (Δ ≤ 1.1°) to the errors in the extreme foot positions (40PF, 20DF), which means that this error is mainly due to STA caused by loading instead of moving the foot. Hence, it is important that the AFM markers are placed in a semi-weightbearing position to minimize this error. The improved validity of the multi-segment foot kinematics due to reducing the effect of STAs also becomes apparent when comparing the AFM output to the kinematics as measured in a bone pin study [54], which is often considered as a gold standard. A similar comparison has been performed for the measured ROMs of OFM and RFM [55]. AFM ROM in the sagittal plane of the hindfoot-shank and forefoot-hindfoot angle (22.7° and 13.5°) are closer to the measured values by bone pins for the calcaneus versus the tibia (17.0°) and the 1st metatarsal versus the talus (17.6°) [54], compared to OFM (27.4° and 10.0°) and RFM (23.8° and 24.3°) [55]. It should be noted that the model evaluation regarding STAs was performed in healthy volunteers. The absolute errors may differ in patient populations for instance due to bony deformities. However, it is not likely that such pathologies would affect what marker locations are most vulnerable to STA, but that remains subject to future studies.

AFM is also more robust to marker misplacements compared to OFM and RFM. In AFM, often the midpoint between markers was used to determine the direction of axes instead of a single marker, to dampen the effect of potential marker misplacement, resulting in a marker placement sensitivity that is roughly half of the OFM and RFM counterparts. Only the sensitivity in the frontal plane of the hindfoot to displacement of the markers on the posterior aspect of the calcaneus was larger than ≥1.0°. This high sensitivity was acceptable as the clinical expert panel considered the varus-valgus angle of the hindfoot as a clinically relevant outcome measure, which can best be described by directly using two markers at the posterior aspect of the calcaneus. Nevertheless, high sensitivity shows the importance of placing these markers as accurately as possible, for example by drawing a line at the back of the calcaneus before placing the markers or using devices that improve the marker placement consistency [56, 57]. The fact that AFM is more robust to marker misplacements potentially improves its repeatability compared to other MFMs, although that obviously also depends on the marker placement accuracy.

The preliminary repeatability analysis of AFM shows acceptable values (i.e. SEM < 5°) for most angular output. Remarkable large variability for AFM was found for the inter-tester repeatability of FF_M_-MF, HX-FF_M_ and TTA, which are all largely dependent on the marker on the basis of the 1st metatarsal. An additional analysis showed that the second tester placed this marker on the dorsal aspect, instead of the instructed dorsomedial aspect of the 1st metatarsal base. Hence the intra-tester variability of these angles was lower (≤3.1^o^) compared to the inter-tester repeatability. Despite these outliers, the median intra- and inter-tester variability over all kinematic temporal patterns of AFM (2.2[0.9°] and 3.1[2.1°]) is still lower or similar to the values reported in repeatability studies of other MFMs. In healthy feet, intra-tester variability values averaged over all angles were reported between 3.9°-4.6° for RFM [20, 21] and 3.0° for the mSHCG foot model [58]. The average inter-tester variability was reported to be between 5.4–7.4° for RFM [20–22] and 3.7^o^ for mSHCG [58]. SEM values were calculated for AFM as well (intra: 3.3 ± 0.9°, inter: 5.2 ± 2.3°), which were lower compared to previously reported intra-tester SEM of OFM (4.61 ± 2.86°) and RFM (3.88 ± 2.18°) [18]. Unlike many of the aforementioned studies we did not report reliability parameters such as ICC, because these are highly dependent on the variability within the studied population, while agreement parameters (e.g. SEM) do not depend on this heterogeneity of the population and are therefore preferred [51]. The current repeatability analysis is limited regarding the number of testers and marker placement sessions and therefore only provides a first indication of the repeatability of AFM. These results suggest a similar, or better, repeatability compared to other foot models,. However, a more extensive repeatability analysis is required, including more testers and measurements sessions, so the variability is not determined over only two measurements. Moreover, the repeatability of AFM should also be determined in clinical populations, including a range of foot deformities.

A novel MFM has to be of additional value to the many models that have already been developed [23]. The previously published MFMs have had a major impact on measuring and understanding foot motion during gait and they serve as the foundation for AFM. Nevertheless, to further develop multi-segment foot kinematic measurements it is important to reduce the substantial and clinically relevant measurement errors that have been shown [24, 30, 34, 36]. AFM is the first MFM that specifically focusses on reducing these measurement errors and thereby improving the measurements. The measurement errors of AFM were only compared to OFM and RFM, which are the MFMs that are among the most frequently used by researchers other than the developers [9], but measurement errors should be quantified for any foot model used in clinical practice or research. Since many models have been developed, reporting standards have been proposed to ensure transparency of methods in MFMs [59]. Therefore, the Matlab code to use AFM and accompanying reference datasets for adults and children have been made available with this paper [53]. This allows others to use and evaluate AFM themselves. The temporal patterns of the main kinematic parameters are also shown in Supplementary file 1.

The clinical applicability of a foot model is at least as important as its validity. Therefore, AFM did not only focus on minimizing measurement errors, but it was also informed by an expert panel, representing a variety in expertise and patient populations. This can be considered a first, yet important step to optimize the clinical utility of the model. So far, limited research has been performed into the clinical efficacy of MFMs and the clinically relevant differences in multi-segment foot kinematics, while these type of studies have been performed for clinical gait analysis in general [52, 60]. Therefore, future studies should evaluate the clinical applicability (e.g. clinical efficacy, impact on treatment decisions or cost effectiveness) of foot models for a range of clinical application scenarios.

The next step for AFM is to be applied in clinical populations. This model was not designed for one specific patient population or age group, but aims to be applicable in a broad range of clinical applications and patient groups. Moreover, it consists of many segments and output parameters. It is important that the complexity of the model and its output align with the clinical or research question of interest. Rankine and colleagues [11] already suggested to use disorder-specific foot models. Instead of developing disorder-specific foot models, we envision to use a subset of segments and/or specific data processing steps to arrive at disorder-specific output of AFM that best fits the clinical problem at hand. Such dedicated output should be developed and evaluated in future studies.

## Conclusion

In this paper we defined the Amsterdam Foot Model, which is a clinically informed multi-segment foot model, that is explicitly developed to minimize kinematic measurement errors. The measurement errors are smaller compared to widely used multi-segment foot models and therefore the model can be considered an improved follow-up to existing models.

## Supplementary Information

**Additional file 1.**

## Data Availability

The datasets used and/or analyzed during the current study are available from the corresponding author on reasonable request. A reference dataset and the Amsterdam Foot Model code are available in an online data repository [51].

## Abbreviations

AFM: Amsterdam Foot Model
OFM: Oxford Foot Model
RFM: Rizzoli Foot Model
MFM: Multi-segment foot model
STAs: Soft tissue artifacts
CS: Coordinate system
CT: Computed tomography
HF-SK: Hindfoot-shank angle
MF-HF: Midfoot-hindfoot angle
FF-HF: Forefoot-hindfoot angle
FF-MF: Forefoot-midfoot angle
FFL-MF: Lateral forefoot-midfoot angle
FFM-MF: Medial forefoot-midfoot angle
HX-FFM: Hallux-medial forefoot angle
F-SK: Foot-shank angle
MLA: Medial longitudinal arch
TTA: Transverse tarsal arch
SD: Standard deviation
SEM: Standard error of measurement
