# Supplementary material for: The Amsterdam Foot Model: a clinically informed multi-segment foot model developed to minimize measurement errors in foot kinematics
Source: J Foot Ankle Res. 2022 Jun 7;15:46. doi: 10.1186/s13047-022-00543-6 (PMC9172122; doi:10.1186/s13047-022-00543-6)
Supplement: Supplementary file 1 — Additional file 1. [file 13047_2022_543_MOESM1_ESM.pdf]

APPENDIX A.

This appendix presents the temporal patterns of the main kinematic parameters of the Amsterdam Foot Model for an adult population. Subject characteristics are provided within the paper in the *Methods – Repeatability* subsection. This reference dataset for adults, a reference dataset for children and the Matlab code of the Amsterdam Foot Model are available for others to use [53].

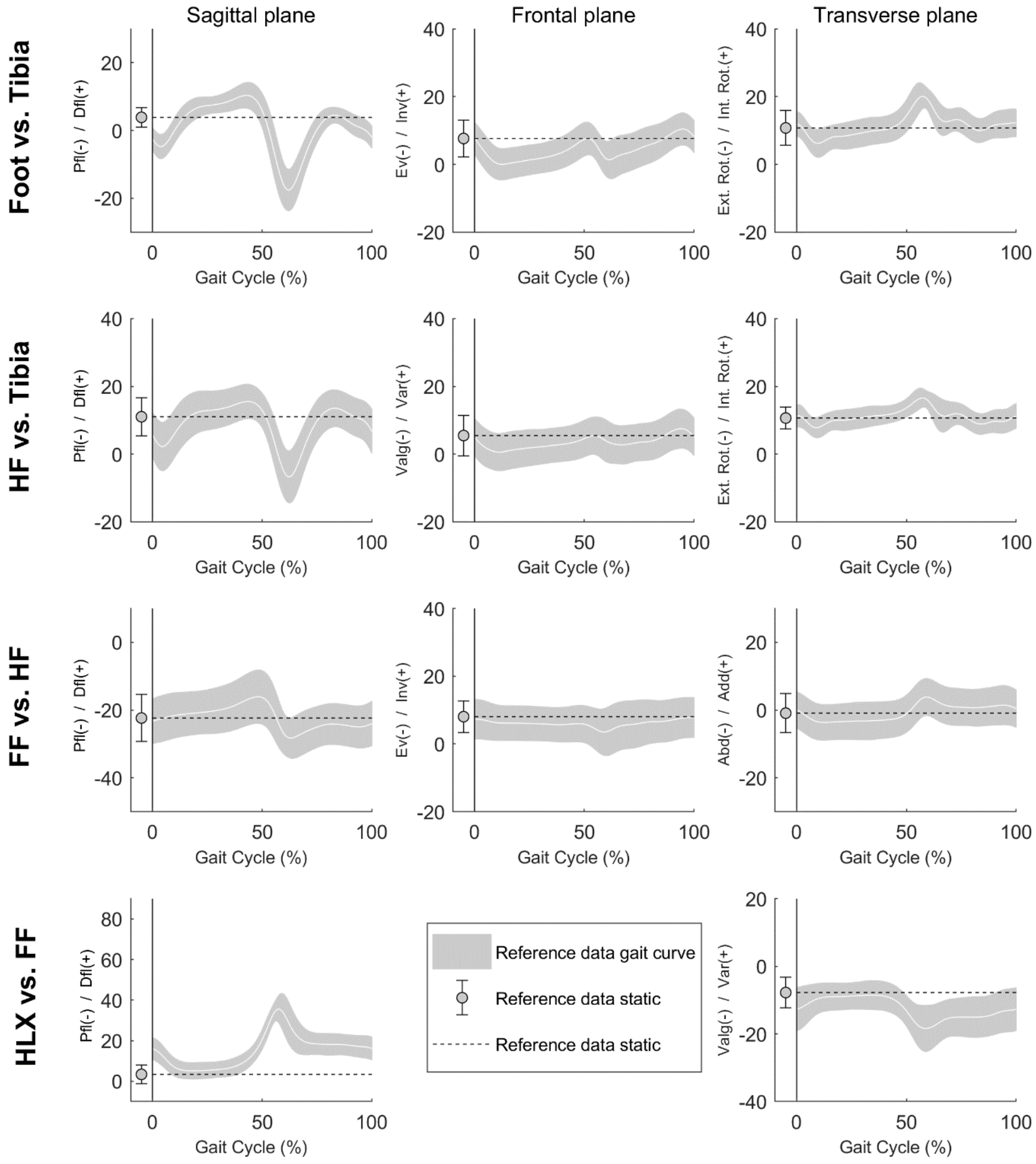

Figure A1. Temporal patterns of a selection of the kinematic parameters of the Amsterdam Foot Model. *Abbreviations: HF: hindfoot, FF: forefoot, HLX: hallux, Pfl: plantarflexion, Dfl: dorsiflexion, Ev: eversion, Inv: inversion, Ext. Rot.: external rotation, Int. Rot.: internal rotation, Abd: abduction, Add: adduction, Valg: valgus, Var: varus.*

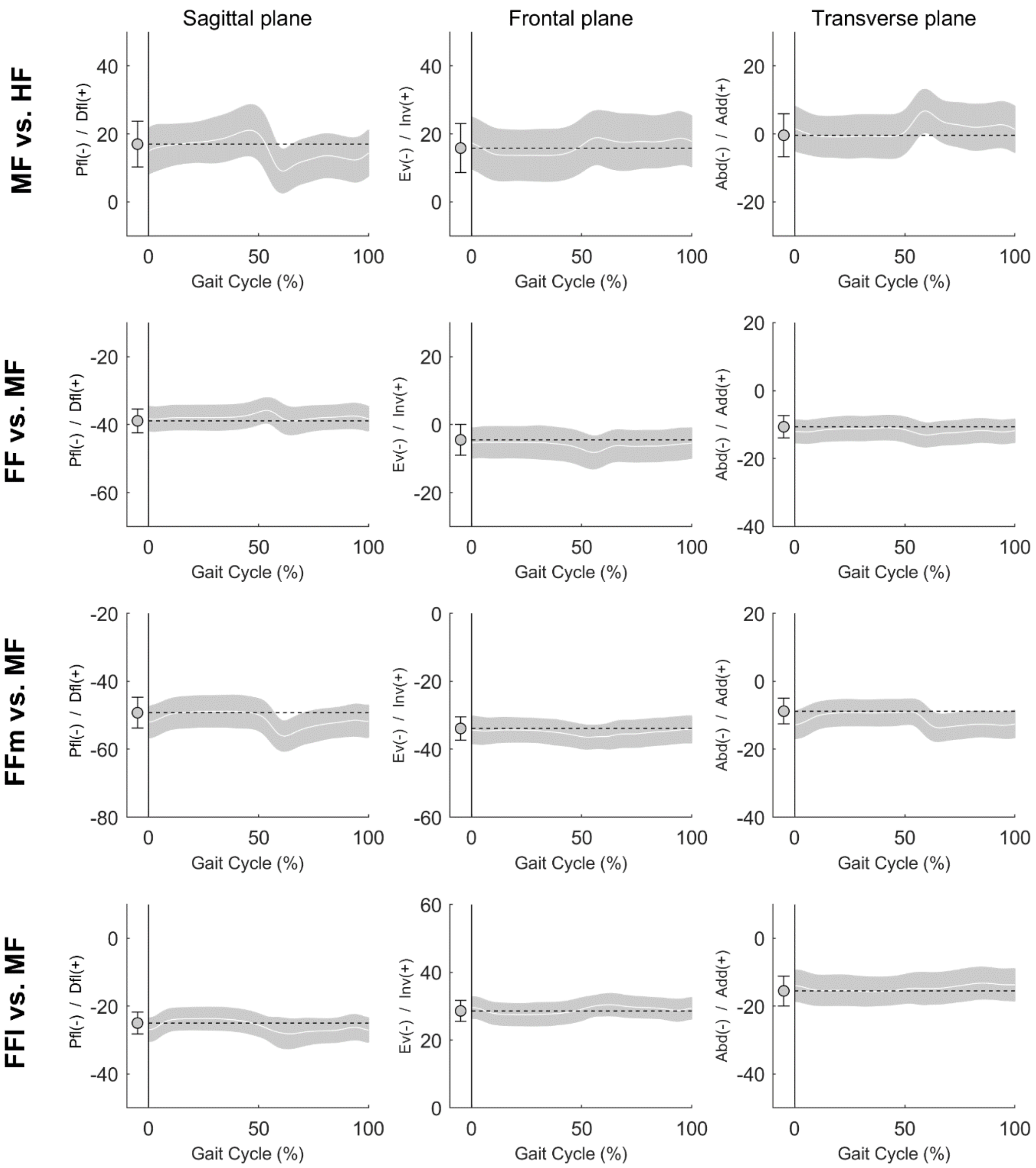

Figure A2. Temporal patterns of a selection of the kinematic parameters of the Amsterdam Foot Model. *Abbreviations: HF: hindfoot, MF: midfoot, FF: forefoot, FFm: medial forefoot, FFl: lateral forefoot, Pfl: plantarflexion, Dfl: dorsiflexion, Ev: eversion, Inv: inversion, Abd: abduction, Add: adduction.*
